# Phage P1-Derived Artificial Chromosomes Facilitate Heterologous Expression of the FK506 Gene Cluster

**DOI:** 10.1371/journal.pone.0069319

**Published:** 2013-07-11

**Authors:** Adam C. Jones, Bertolt Gust, Andreas Kulik, Lutz Heide, Mark J. Buttner, Mervyn J. Bibb

**Affiliations:** 1 Pharmaceutical Institute, University of Tübingen, Tübingen, Germany; 2 Department of Microbiology and Biotechnology, University of Tübingen, Tübingen, Germany; 3 Department of Molecular Microbiology, John Innes Centre, Norwich Research Park, Norwich, United Kingdom; University Paris South, France

## Abstract

We describe a procedure for the conjugative transfer of phage P1-derived Artificial Chromosome (PAC) library clones containing large natural product gene clusters (≥70 kilobases) to *Streptomyces coelicolor* strains that have been engineered for improved heterologous production of natural products. This approach is demonstrated using the gene cluster for FK506 (tacrolimus), a clinically important immunosuppressant of high commercial value. The entire 83.5 kb FK506 gene cluster from *Streptomyces tsukubaensis* NRRL 18488 present in one 130 kb PAC clone was introduced into four different *S. coelicolor* derivatives and all produced FK506 and smaller amounts of the related compound FK520. FK506 yields were increased by approximately five-fold (from 1.2 mg L^-1^ to 5.5 mg L^-1^) in *S. coelicolor* M1146 containing the FK506 PAC upon over-expression of the FK506 LuxR regulatory gene *fkbN.* The PAC-based gene cluster conjugation methodology described here provides a tractable means to evaluate and manipulate FK506 biosynthesis and is readily applicable to other large gene clusters encoding natural products of interest to medicine, agriculture and biotechnology.

## Introduction

A wealth of microbial gene clusters that encode the biosynthetic pathways to bioactive natural products, also known as secondary or specialized metabolites, are being unveiled with the ever-increasing number of sequenced bacterial genomes [1]. While some clusters correspond to known compounds, a large number (so-called cryptic gene clusters) encode the biosynthesis of previously undiscovered molecules and represent a promising source of new drugs and therapeutics. Many of these gene clusters feature modular type I polyketide synthase (PKS) and/or non-ribosomal peptide synthetase (NRPS) enzymes, and can exceed 100 kilobases (kb) in size and consist of several tens of genes [2], [3].

One of the most useful strategies for advancing the study of natural products is heterologous expression of the genes responsible for biosynthesis in a tractable host organism. This approach has many advantages, including compound yield improvements [4], [5], [6] and the ability to work with a strain that is more amenable to genetic manipulation, e. g. in order to disrupt genes [7], to create analogs [8], to study regulation [9], to determine the minimal gene set necessary for biosynthesis [10], or to aid in the identification of unknown molecules, such as from metagenomic libraries [11], [12].

While heterologous expression has been used successfully with small biosynthetic gene clusters (≤40 kb) [13], standard techniques are not as straightforward with large biosynthetic gene clusters (≥70 kb), largely because of the amount of DNA that needs to be cloned and transferred into a suitable expression host. In frequently used *E. coli* cosmid libraries, insert sizes are limited to approximately 42 kb. Thus, large clusters are typically fragmented across multiple clones and require often laborious reassembly strategies such as Red/ET-mediated recombination in *E. coli* [7], [14] or Transformation Associated Recombination (TAR) in yeast [12], [15].

One method that avoids most problems with cluster reassembly is to clone large gene clusters directly into artificial chromosomes that are able to tolerate insert sizes of approximately 200 kb [16], [17]. This size capacity is sufficient for virtually all known bacterial modular gene clusters and should also allow for several kb of adjacent DNA sequence to be included if the cluster boundary regions are unclear. However, techniques for subsequent handling of these artificial chromosomes and their introduction into heterologous expression strains are not always straightforward. Vectors with large inserts that require protoplast transformation [6], [18] are prone to sequence deletions or rearrangements and frequently give very low transformation rates.

In this paper, we provide an expedient, reliable procedure for transfer of large natural product gene clusters using phage P1-derived Artificial Chromosomes (PACs) to *Streptomyces coelicolor* derivatives that have been engineered for improved heterologous expression. This approach allows for stable integration of PAC DNA via conjugation without any additional modifications, immediately creates a platform for gene cluster analysis, and enables targeted systems and synthetic biology approaches that might not be possible in wild-type strains. To demonstrate the feasibility of this procedure, we have heterologously expressed the 83.5 kb biosynthetic gene cluster for FK506 (tacrolimus), a clinically important polyketide from *Streptomyces tsukubaensis* NRRL 18488, using a PAC construct of approximately 130 kb.

FK506 is a high value calcineurin inhibiting immunosuppressant [19], marketed under the trade name Prograf with annual sales of approximately $2 billion (evaluatepharma.com, [20]). It is now used following bone marrow, kidney and liver transplants [21], [22], [23], is effective at treating skin inflammation and eczema [24], [25] and is gaining additional attention for its potential in treating cancer [26] and neurological conditions [27].

**Figure 1:**
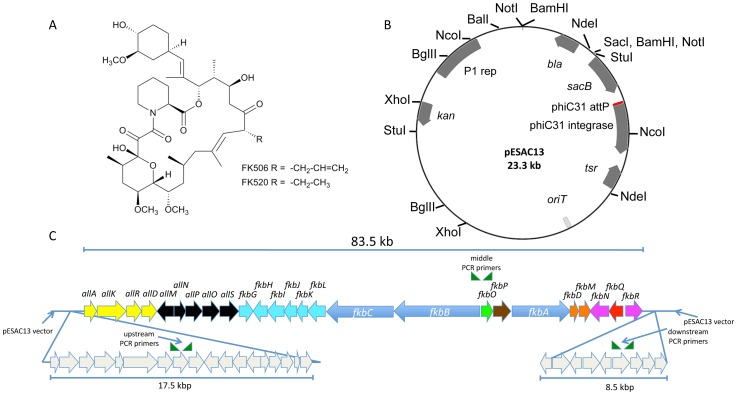
Phage P1-derived Artificial Chromosome (PAC)-based approach for heterologous expression of FK506. A) Structures of FK506 and FK520. B) Vector map of PAC pESAC13. C) Region of *Streptomyces tsukubaensis* NRRL 18488 genome included in PAC20N and used for heterologous expression. Gene colors refer to the following functions [30]: Yellow, allylmalonyl-CoA biosynthesis; light blue, methoxymalonyl-ACP biosynthesis; dark blue, polyketide synthase; green, chorismate hydrolase (starter unit biosynthesis); brown, peptide synthetase; orange, post-PKS modification; purple, FK506 regulation; red, thioesterase; black, other genes.

Both FK506 and the related natural product FK520 (ascomycin) are produced by several *Streptomyces* strains. The two molecules differ only in one position with the presence of either an allyl (FK506) or ethyl (FK520) side chain (Figure 1A). Several recent studies have greatly expanded our understanding of FK506 and FK520 biosynthesis, including the identification of the enzyme FkbO as a chorismate hydrolase that generates the first FK506/FK520 precursor [28], the enzymes responsible for constructing allylmalonyl CoA or ethylmalonyl CoA for the two different side chains of FK506 and FK520 [29], [30], [31] and genes whose products positively or negatively regulate FK506 expression [32], [33]. Introduction of precursors into culture media or over-expression of certain components of the FK506 gene cluster have also led to improvements in FK506 yields [34], [35]. Despite these advances, several challenges exist in pursuing FK506 yield improvements in wild-type *Streptomyces* producers. Genomic information for FK506 producing *Streptomyces* has only recently emerged [36], and targeted genetic manipulations in some producing strains are hindered by slow growth or difficulties with introduction of exogenous DNA. In addition, FK506 production is sensitive to a number of culture conditions including pH and the concentrations of lysine, nitrogen, and phosphate [20].

These challenges made FK506 an ideal candidate molecule for heterologous production via PAC based conjugation. Production of FK506 in *S. coelicolor* should enhance the ability to make targeted gene replacements or modifications and potentially provide new avenues for industrial production.

## Results

### Generation of a PAC library using the P1-derived artificial chromosome pESAC13

For the generation of a genomic library of *S. tsukubaensis* NRRL 18488, we used the phage P1-derived artificial chromosome vector ( = PAC vector) pESAC13, depicted in Figure 1B. This vector was developed by M. Sosio and S. Donadio, NAICONS, Milano, Italy and is used by Bio S&T Inc. (Montreal, Canada; see below) for PAC library construction. pESAC13 contains the phage P1 origin of replication, as well as the phage phiC31 integration system that allows stable insertion into the genomes of most *Streptomyces* species. In contrast to previously developed *E. coli*-*Streptomyces* Artificial Chromosomes, pESAC13 (a derivative of pPAC-S1, [16]) contains an *oriT* site that allows transfer into *Streptomyces* by conjugation, rather than by time-consuming protoplast transformation. The *bla* resistance gene in pESAC13 is lost upon library construction; however, the vector also confers kanamycin resistance in *E. coli* and thiostrepton resistance in *Streptomyces*.

A major technical challenge in the generation of large insert libraries is the development of protocols for the isolation of high-molecular weight DNA, especially from uncharacterized actinomycetes where even cell lysis can be problematic. This can now be outsourced to companies specializing in this technique; in our case, Bio S&T Inc. (Montreal, Canada; www.biost.com). Starting from 7 ml flash-frozen packed mycelium, high-molecular weight DNA was isolated by Bio S&T and cloned between the two BamHI sites of pESAC13, which results in the loss of carbenicillin resistance from the parent vector (Figure 1B). A library of 1920 PAC clones was generated.

### Identification of PAC clones containing the complete FK506 gene cluster

Comparison of the previously published sequences of the FK506 biosynthetic gene clusters from different producer strains [30], [36] confirmed that all known FK506 clusters contain a core set of genes including *allA*-*allD*, the three PKS genes *fkbA*-*fkbC*, and *fkbG*-*fkbQ*, with certain strains also containing orthologs of the genes *allMNPOS* and the regulatory gene *fkbR* (Figure 1C). All of these genes are present in an 83.5 kb region of the *S. tsukubaensis* genome. This region therefore contains all genes known to be involved in FK506 biosynthesis [29], [30] as well as *allMNPOS*, whose functions have not been elucidated. The recently published genome sequence of *S. tsukubaensis* NRRL 18488 [36] allowed for bioinformatic investigation of the DNA regions that flank this core cluster. On the left side of the cluster depicted in Figure 1C a putative NRPS siderophore gene cluster was identified that contained putative regulatory genes, while on the right side no genes were identified with any obvious role in secondary metabolite biosynthesis.

The *S. tsukubaensis* PAC library was screened with the three primer pairs listed in Table 1 and shown in Figure 1C, amplifying fragments flanking and within the cluster. Screening of the first 768 PAC clones ( =  two 384 well plates) resulted in two clones showing amplification with all three primer pairs. Gel electrophoresis suggested the inserts to be 160 and 110 kb in size, respectively. Both of these clones were end-sequenced, confirming that they spanned the DNA region containing the FK506 cluster. The smaller PAC, designated 20N, contained the 109347 bp insert depicted in Figure 1C and was chosen for heterologous expression studies.

**Table 1:** Bacterial strains, vectors, and PCR primers used in this study.

Bacterial strains	Reference
*Streptomyces tsukubaensis* NRRL 18488	US Department of Agriculture, [36]
*Streptomyces coelicolor* M512	[40]
*S. coelicolor* M1146	[5]
*S. coelicolor* M1152	[5]
*S. coelicolor* M1154	[5]
*E. coli* ET12567	[43]

Vectors	Function, reference
pESAC13	Phage Artificial Chromosome (PAC) vector (Margherita Sosio, NAICONS).
pUWL-*oriT*-*apr* containing ermE* promoter	Protein expression vector, [44]
pR9406	Driver plasmid (David Figurski)

PCR primers	Primer sequence (restriction sites are underlined and ribosome binding sites [54] are in bold text)
FK506 upstream forward screening primer	CGGGCCGGCGCAAGCAGCCGAACT
FK506 upstream reverse screening primer	CGGCCACACCGGACAGGACCAGGCG
FK506 middle forward screening primer	CGGCGAGGTGACCGGCGATCCGGC
FK506 middle reverse screening primer	GCGGCAGTACGGTGCCGCGACAGGC
FK506 downstream forward screening primer	ACCGGGAGCGGCGCGACACCATGC
FK506 downstream reverse screening primer	TCATGCCTGGTCGGGGCCGGGTGCC
*fkbN* RBS HindIII forward primer	GGGGAAGCTT **AGCAACGGAGG TACGGAC**GTGGTTCCGGAAGTGCG AGCAG
*fkbN* SpeI reverse primer	GGGGACTAGTCTACCCGCACCGGT CGCCCCGG
*fkbR* RBS SpeI forward primer	GGGGACTAGT **AGCAACGGAGGTAC GGAC**ATGTTTCGACGTTTTTCTGC
*fkbR* SpeI reverse primer	GGGGACTAGTTCAGTCCTGTGGGGCG

### Integration of the FK506 gene cluster into the genomes of engineered *S. coelicolor* host strains

*S. coelicolor* is the genetically best-characterized streptomycete, and a large number of tools are available for its genetic manipulation [37], [38], [39]. *S. coelicolor* M512 [40] has been used previously as a heterologous expression host [13]. Recently, M1146 was developed as an improved host; it lacks all of the genes required for the production of actinorhodin, the prodiginines, coelimycin, methylenomycin and the calcium-dependent antibiotic, and thus lacks anti-microbial activity. The deletion of these biosynthetic gene clusters also removes unwanted sinks of carbon and nitrogen, and hence competition for precursors, and prevents potential cross-talk between different biosynthetic pathways that might result in product inactivation [5]. The strain also has a much simplified extracellular metabolite profile, greatly facilitating analysis of culture supernatants by mass spectrometry. Strains M1152 and M1154 both carry additional mutations in RNA polymerase (in the RpoB subunit) and M1154 also has a mutation in ribosomal protein S12. Both mutations were reported previously to increase the production levels of several secondary metabolites [17], [41], [42].

**Figure 2:**
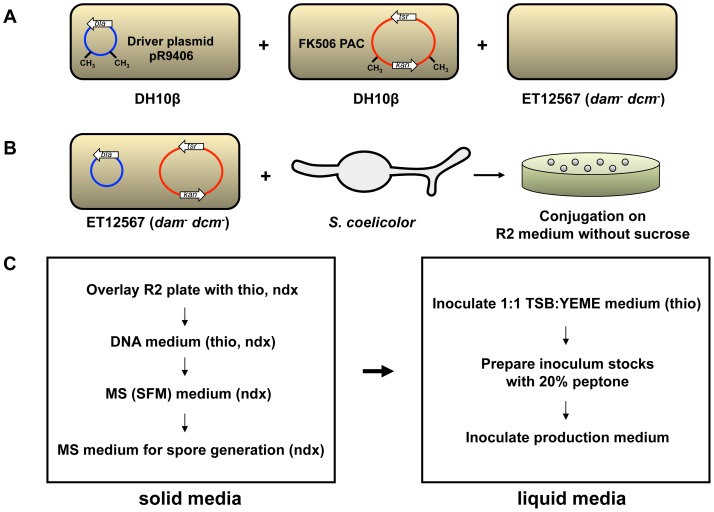
Procedure for introduction of PAC DNA into *Streptomyces coelicolor*. A) Two DH10β strains, containing either the pR9406 helper plasmid (carbenicillin resistance) or the FK506 PAC (kanamycin resistance in *E. coli*, thiostrepton resistance in *S. coelicolor*), are triparentally mated on LB agar without antibiotics with the non-methylating *dam* ^-^ *dcm* ^-^ ET12567 strain (chloramphenicol resistance). Following the mating, the resultant *E. coli* patch is restreaked to select for ET12567 cells that contain both vectors. B) Resistant colonies are grown to an OD_600_ of 0.4, conjugated with *S. coelicolor* spores and plated on R2 medium without sucrose [37]. C) Additional plating and inoculation steps to prepare *S. coelicolor* for production medium. thio  =  thiostrepton, ndx  =  nalidixic acid.

PAC clone 20N containing the FK506 cluster (PAC20N) as well as a corresponding empty pESAC13 vector (pESAC13*Δbla*; see Materials and Methods) used as a control were moved into the non-methylating *E.coli* ET12567 strain [43] via triparental mating and introduced into *S. coelicolor* M512, M1146, M1152 and M1154 by conjugation, following the procedure summarized in Figure 2.

### Heterologous production of FK506

**Figure 3:**
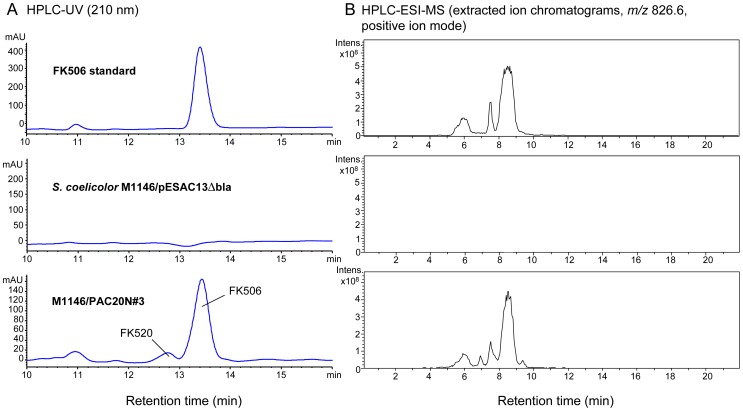
Heterologous expression of FK506 in *Streptomyces coelicolor* M1146. A) HPLC-UV chromatograms showing production of FK506 in *S. coelicolor* M1146 after introduction of PAC20N containing the FK506 cluster. No production is observed upon introducing an empty pESAC13 vector. B) Extracted ion chromatograms of separate HPLC-ESI-MS analyses corresponding to the adjacent HPLC-UV data, verifying the presence or absence of FK506 in these strains.

Upon cultivation in MGm 2.5 medium [20], production of FK506 was detected in all strains containing PAC20N using HPLC-UV and HPLC-MS in comparison to an authentic FK506 standard (Inresa Pharma, Bartenheim, France). Figure 3 shows representative chromatograms from strain M1146/PAC20N. Figure S1 shows confirmation of the identity of FK506 by LCMS-MS analysis. No FK506 was detected after the introduction of pESAC13Δ*bla*.

**Figure 4:**
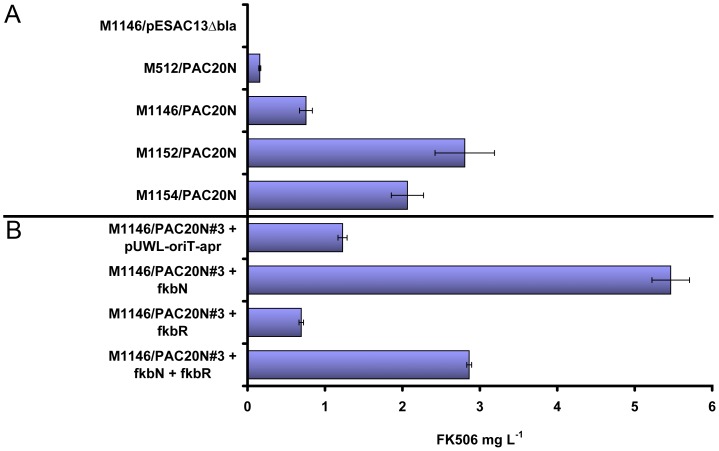
FK506 production yields in *Streptomyces coelicolor* strains. (A) Comparison of average FK506 production yields in different *S. coelicolor* host strains. The number of independent exconjugants assayed from each strain was: For M1146/pESAC*Δbla*, N = 1; for M512/PAC20N and M1154/PAC20N, N = 2; for M1146/PAC20N and M1152/PAC20N, N = 3. Each exconjugant was assayed in triplicate in parallel cultivations in MGm 2.5 media in microtiter plates [52]. Data show mean values and standard errors of all measurements. (B) Effect of over-expression of the regulator genes *fkbN* and *fkbR* on FK506 production in *S. coelicolor* M1146/PAC20N#3 as compared to M1146/PAC20N#3 containing an empty pUWL-*oriT*-*apr* vector. Averages from the highest producing exconjugants from assays testing M1146/PAC20N#3 + *fkbN*, + *fkbR* and *fkbN* + *fkbR* are shown.

Average production levels in exconjugants from strains M1146, M1152 and M1154 were 0.75, 2.81 and 2.06 mg L^−1^, respectively (Figure 4A), clearly exceeding production in M512 (0.16 mg L^−1^). As observed previously [4], production levels varied among independent exconjugants (e.g. 0.53, 0.68 and 1.05 mg L^−1^ for three exconjugants of M1146) but were reproducible in repeated cultivations of the same exconjugant strain. A smaller amount of the related compound FK520 was also produced in the strains containing PAC20N, amounting to approximately 10% of FK506 production (Figure 3). This FK506∶FK520 production ratio was comparable to that observed in *Streptomyces tsukubaensis* NRRL 18488 under the same growth conditions.

Growth rate and production were most reproducible in strain M1146, therefore *S. coelicolor* M1146/PAC20N exconjugant #3 (1.05 mg L^−1^) was chosen for subsequent experiments to determine if FK506 yields in *S. coelicolor* could be improved by over-expression of FK506 regulatory genes.

### Over-expression of *fkbN* increases FK506 production levels

FK506 production in wild-type *Streptomyces* strains appears to be positively regulated primarily by the LuxR-type transcriptional regulator FkbN. Over-expression of *fkbN* increased FK506 yields in *S. tsukubaensis* NRRL 18488 [33] and *Streptomyces* sp. KCTC 11604BP [32] and *fkbN* is present in all other identified FK506 gene clusters [30]. We introduced *fkbN* into S. *coelicolor* M1146/PAC20N#3 using the replicative, conjugative expression plasmid pUWL-*oriT*-*apr* containing the strong constitutive ermE* promoter [44] and monitored FK506 production levels as above. While growth rates and biomass accumulation were comparable, over-expression of *fkbN* resulted in approximately five-fold FK506 yield improvements (5.5 mg L^−1^ compared to 1.2 mg L^−1^ observed upon introduction of an empty pUWL-*oriT*-*apr* plasmid; Figure 4B), demonstrating that *fkbN* over-expression is also beneficial to FK506 yields when the cluster is expressed heterologously.

The LysR-type regulator FkbR has also been shown to influence FK506 production levels, although this gene or its homologues have not been observed in all FK506 gene clusters [30]. In *Streptomyces* sp. KCTC 11604BP, over-expression of the *fkbR* homologue *tcs7* decreased FK506 production, while *tcs7* deletion elevated FK506 yields [32]. In contrast, in *S. tsukubaensis* NRRL 18488 over-expression of *fkbR* increased FK506 production levels, while deletion had the opposite effect [33]. We introduced *fkbR* into *S. coelicolor* M1146/PAC20N#3 in the same manner as *fkbN*, but observed a decrease of 44% (0.69 mg L^−1^, Figure 4B). It appears that constitutive, high *fkbR* expression in *S. coelicolor* is detrimental to FK506 yields.

We also generated a pUWL-*oriT*-*apr* expression plasmid containing tandemly arranged *fkbN* and *fkbR* (each with the same ribosome binding site that was used for their individual expression) that resulted in an increase of FK506 production (2.86 mg L^−1^), but clearly less than the highest yields obtained when over-expressing *fkbN* alone (Figure 4B). This is consistent with the positive regulatory effect of *fkbN* over-expression being counteracted by a negative effect of constitutive expression of *fkbR*.

## Discussion

FK506 (tacrolimus) is the most important immunosuppressant in clinical use [45]. Substantial effort has led to yield improvements in wild-type producers through regulator manipulation [32], [33], precursor supplementation into culture media [31], [34], [46] or by introducing extra copies of FK506 biosynthetic enzymes [35]. To the best of our knowledge, our PAC-based conjugation strategy has afforded the first example of heterologous FK506 production in a more tractable expression host. Transfer of the FK506 gene cluster to *S. coelicolor* removes any obstacles inherent in working with native producer strains, including issues with sporulation, suitable growth media, genetic instability, or genetic manipulation. Additionally, while genetic modifications in wild-type strains require time-consuming double crossover experiments, PAC introduction into heterologous strains such as *S. coelicolor* permits rapid modification of the gene cluster in *E. coli* and direct examination of the primary exconjugants in the heterologous host, thereby reducing the amount of steps needed for such investigations. It also allows use of the wide variety of genetic tools available for *S. coelicolor*. Production of FK506 in *S. coelicolor* confirms that all genes necessary for its synthesis in a heterologous streptomycete host are present in our PAC clone, which simplifies future analysis of FK506 and precursor biosynthesis.

Although initial FK506 yields in *S. coelicolor* M1146 were modest (up to approximately 1 mg L^−1^ in microtiter plates, Figure 4) significant yield improvements were observed upon over-expression of the positive FK506 regulator *fkbN* (5.5 mg L^−1^) and we anticipate additional modifications should continue to increase heterologous production yields. However, FK506 production decreased upon over-expression of *fkbR* in *S. coelicolor* M1146/PAC20N#3, which is contrary to recent evidence showing the positive effect of *fkbR* on FK506 production levels in *S. tsukubaensis* NRRL 18488 [33]. This result may indicate that *fkbR* functions differently in *S. coelicolor*, perhaps by affecting an unknown component of global regulation that has some influence on FK506 biosynthesis. It is also possible that high level expression of *fkbR* is detrimental to FK506 biosynthesis, and that this regulator might normally be transcribed at lower levels and/or in a time-controlled fashion. This finding may be reflected in the opposing conclusions reported in other studies of *fkbR* [32], [33].

The triparental mating and conjugation procedure we describe here for stable transfer of PAC DNA should be applicable to many other natural product gene clusters of biomedical and/or biotechnological interest. Actinobacteria are among the most prolific microbial producers of natural products and the majority of known antibiotics are derived from them [47], [48]. Several antibiotics from actinobacteria are encoded by large gene clusters such as enduracidin (84 kb, [49]), and rubradirin (105 kb, [2]), and could also be useful targets for heterologous expression via PAC cloning. With increasing amounts of data available from genome sequencing projects, PAC-based strategies hold significant value and promise in simplifying subsequent biosynthetic investigations. These methods may be especially valuable when large unidentified gene clusters appear to be “silent”, only produce trace amounts of compound that complicate or prohibit structural elucidation, or are found in rare or intractable bacterial strains that do not have any genetic techniques established. In addition, PAC-based conjugation could serve as a means of mobilizing large artificially derived natural product gene clusters as gene synthesis technologies become more affordable. Future work with the PAC containing the FK506 gene cluster or with other PACs containing gene clusters of high relevance could also be targeted at improving industrial compound yields by testing a variety of heterologous hosts and eventually lowering production costs.

We envision that large insert DNA libraries will become a standard complement to microbial genome sequencing projects in the near future [6]. The amount of available sequence information outpaces experimental verification of gene function, and although the quality of bioinformatics-based prediction of natural product gene clusters has advanced tremendously over the last decade [50] strategies to accelerate gene cluster identification and structure elucidation and increase compound production are urgently needed. The PAC based methodology described here to mobilize natural product gene clusters of virtually any size to engineered heterologous *Streptomyces* production strains should contribute to shortening this timeline and remove impediments to genetic or system biology studies aimed at improving access to compounds of interest.

## Materials and Methods

### Antibiotic selection

Apramycin (50 µg ml^−1^), carbenicillin (50 µg ml^−1^), kanamycin (50 µg ml^−1^), chloramphenicol (25 µg ml^−1^), nalidixic acid (25 µg ml^−1^) and thiostrepton (10 µg ml^−1^ (liquid media) or 60 µg ml^−1^ (plates)) were added to growth media for appropriate selection as required unless otherwise noted.

### Preparation of Phage P1 Artificial Chromosome (PAC) library

*Streptomyces tsukubaensis* NRRL 18488 was obtained from the US Department of Agriculture strain collection and grown on ISP4 plates [20]. Mycelia were prepared for PAC library construction by growing *S. tsukubaensis* in YEME medium (100 ml) inoculated with mycelia from a TSB starter culture (10 ml, [37]). The mycelia were grown in YEME for 72 h at 30°C in a 300 ml baffled flask with a stainless steel spring, and were then centrifuged to remove the media. The mycelial pellets were washed three times (200 mM NaCl, 10 mM Tris-Cl (pH 7.2), 100 mM EDTA (pH 8.0)) and flash frozen in liquid nitrogen before being stored at −80°C. PCR primers (Table 1) were designed for PAC library screening to isolate positive clones containing the entire FK506 gene cluster, and were tested beforehand using *S. tsukubaensis* genomic DNA as PCR template. Each PCR product was sequenced to verify that the intended regions of the gene cluster were amplified. Approximately 7 ml of concentrated mycelia were used to construct a phage P-1 artificial chromosome library using a modified *E. coli – Streptomyces* Artificial Chromosome (ESAC, [16]) vector (pESAC13, Figure 1B). This vector contains an *oriT*, a phiC31 integrase gene and a phiC31 attP site that permit conjugation into *Streptomyces* strains and integration at the attB recombination locus.

The pESAC13 library construction was performed by Bio S&T (Montreal, Canada). Vector DNA was digested with BamHI, dephosphorylated and purified using standard procedures. High-molecular-weight (HMW) DNA was isolated by embedding the mycelium in 1% low-melting-point agarose plugs that were treated with proteinase K overnight, and then partially-digested with Sau3AI. The partially digested HMW DNA was size-fractionated on a 1% (w/v) pulsed field agarose gel in 0.5X TBE using a CHEF DRIII (Bio-Rad, Canada). The 100–250 kb DNA fragments were eluted from the gel by PFGE and dialyzed against 1X TE (10 mM Tris-HCl, 1 mM EDTA, (pH 8.0)) prior to ligation with the vector. The ligation mix was used to transform 20 µl of *E. coli* DH10β cells (Invitrogen, USA) by electroporation using a CellPorator equipped with a voltage booster (Invitrogen, USA). Transformants were selected at 37°C on LB medium supplemented with 20 µg ml^−1^ kanamycin and 5% sucrose. For quality control, insert size was determined by DraI or NotI digestion of mini-prepped PAC DNA and subsequent PFGE gel separation. The average insert size of the pESAC library was estimated to be 125 kb.

Two PACs (approximated by gel electrophoresis to contain inserts of 160 and 110 kb) were identified by PCR screening as likely to contain the entire FK506 gene cluster. Both PACs were end sequenced, and the smaller of the two PACS (PAC20N, determined by end sequencing to contain a 109347 bp insert) was chosen for heterologous expression (Figure 1C). The DH10β *E. coli* strain containing PAC20N was used in a triparental mating (Figure 2) with another DH10β *E. coli* strain that contained the driver plasmid pR9406 (derived from pUB307 [51]), in which the kanamycin marker has been replaced with carbenicillin) and the non-methylating *E. coli* strain ET12567. Using overnight starter cultures, these three strains were grown to an OD_600_ of 0.4 and mixed together on LB plates without antibiotic selection. After 24 h, the resulting *E. coli* patch was streaked on selective (kan^R^, carb^R^, chl^R^) LB plates. The resulting ET12567 exconjugants were tested by PCR to confirm that they contained the entire PAC20N clone insert. To create a negative control strain to use in parallel to the PAC20N clone, pESAC13 was digested with BamHI and ScaI and religated to remove the *bla* resistance gene. This empty vector, pESAC13*Δbla*, was also transferred to ET12567 by triparental mating for later conjugation into *S. coelicolor*.

### Introduction of PACs into *S. coelicolor* strains

The ET12567 strains containing pR9406 and either PAC20N or pESAC13*Δbla* were conjugated with germlings of *S. coelicolor* M512, M1146, M1152, and M1154 [5] according to standard protocols [37] (Figure 2). Following conjugation, mixtures of spores and *E. coli* were plated on R2 medium without sucrose and overlaid after 16–20 h with thiostrepton (60 µg ml^−1^) and nalidixic acid (25 µg ml^−1^). Exconjugants were streaked on DNA plates containing thiostrepton and nalidixic acid at the same concentrations, and then on MS (SFM) plates with 10 mM MgCl_2_ and nalidixic acid to allow colonies to sporulate. Three sporulating colonies from each *S. coelicolor* strain were spread on new MS+MgCl_2_+ nalidixic acid plates and allowed to grow for 5–7 d prior to harvesting spores for inoculating cultures. After harvesting, dilution series of spores were spread on MS plates to obtain concentrations for each spore stock.

Spores (5×10^8^) of each strain were inoculated into 50 ml 1∶1 TSB:YEME cultures containing thiostrepton (10 µg ml^−1^). Each culture was grown for 3 d at 30°C in baffled flasks as described above. The mycelia were collected and frozen overnight in 20% peptone (7.5 ml total volume in 50 ml Falcon tubes). The mycelia were subsequently homogenized [52] and stored at −80°C to serve as stocks for inoculation of production medium. To measure heterologous production of FK506, each strain was grown in 24-well microtiter plates (28°, 300 RPM, [4], [52]) for 6 days in MGm 2.5 medium ([20] with minor modification). Each well of the 24-well microtiter plate contained 3 ml of MGm 2.5 media and was inoculated with 75 µl of homogenized mycelia (3 wells per culture replicate). For quantification of FK506 production in each *S. coelicolor* strain, PAC20N and pESAC13Δ*bla* integrants were grown in triplicate.

### Detection of FK506 and FK520

Culture samples from each *S. coelicolor* strain were extracted with ethyl acetate (1∶1) and each extract was reduced to dryness using a speed vacuum system. Samples were resuspended in 100 µl of methanol and profiled using HPLC (25 µl injections) and LCMS (2.5 µl injections) in comparison to standards of FK506 (Inresa Pharma, Bartenheim, France) and FK520 (Sigma). An HPLC solvent system of A) H_2_O with 0.01% TFA and B) 20% methyl tert-butyl ether (MTBE) in acetonitrile (ACN) was used to resolve the FK520 and FK506 peaks (43%–100% B∶A over 35 min, 0.4 ml min^−1^, 55°C) using a ReproSil-Pur Basic C18-HD, 3 µm, 150×3 mm HPLC column (Dr. Maisch GmbH, Ammerbuch-Entringen, Germany). LCMS analysis of these samples was performed with an Agilent HPLC-ESI-MS system (LC/MSD Ultra Trap System XCT 6330, Waldbronn, Germany), using a gradient of A) H_2_O with 0.1% formic acid and B) 0.06% formic acid in ACN (50%–100% B∶A over 20 min, 100% B to 22 min, 0.4 ml min^−1^, 40°C) on a Nucleosil 100 C18 3 µm column (100×2 mm ID) with a precolumn (10×2 mm ID) (Dr. Maisch). Detection of *m/z* values consistent with FK506 and FK520 was conducted with Agilent DataAnalysis for 6300 Series Ion Trap LC/MS 6.1 ver. 3.4 software (Bruker-Daltonik GmbH).

### Genetic modifications to *S. coelicolor* M1146 containing the FK506 PAC

The genes encoding the FK506 regulators FkbN and FkbR were PCR amplified from the FK506 PAC and ligated independently into separate pUWL-*oriT-apr* vectors containing the ermE* promoter [44], [53]. The forward primer for each amplicon (Table 1) contained a *Streptomyces* ribosome binding site [54]. These constructs were verified by sequencing and conjugated, along with pUWL-*oriT-apr* as a control, into *S. coelicolor* M1146/PAC20N exconjugant #3 using standard protocols [37], [43]. This *S. coelicolor* M1146 FK506 derivative had shown the highest levels of FK506 production when assaying *S. coelicolor* M1146/PAC20N exconjugants in preliminary experiments. Another pUWL-*oriT-apr* construct was created containing both *fkbN* and *fkbR*, with each gene preceded by the same ribosome binding site as above [54]. All plates, preculture media and production media used for growing *S. coelicolor* strains containing the pUWL-*oriT-apr* constructs were maintained under apramycin selection.

## Supporting Information

Figure S1 **LCMS-MS analysis of FK506 standard (top) and FK506 produced by** ***Streptomyces coelicolor*** **M1146/PAC20N#3 (bottom).** The parent ion is the sodium adduct of FK506 (826.7 *m/z*).
(TIF)
