# Supplementary figures and images for: Phage P1-Derived Artificial Chromosomes Facilitate Heterologous Expression of the FK506 Gene Cluster
Source: PLoS One. 2013 Jul 11;8(7):e69319. doi: 10.1371/journal.pone.0069319 (PMC3708917; doi:10.1371/journal.pone.0069319)

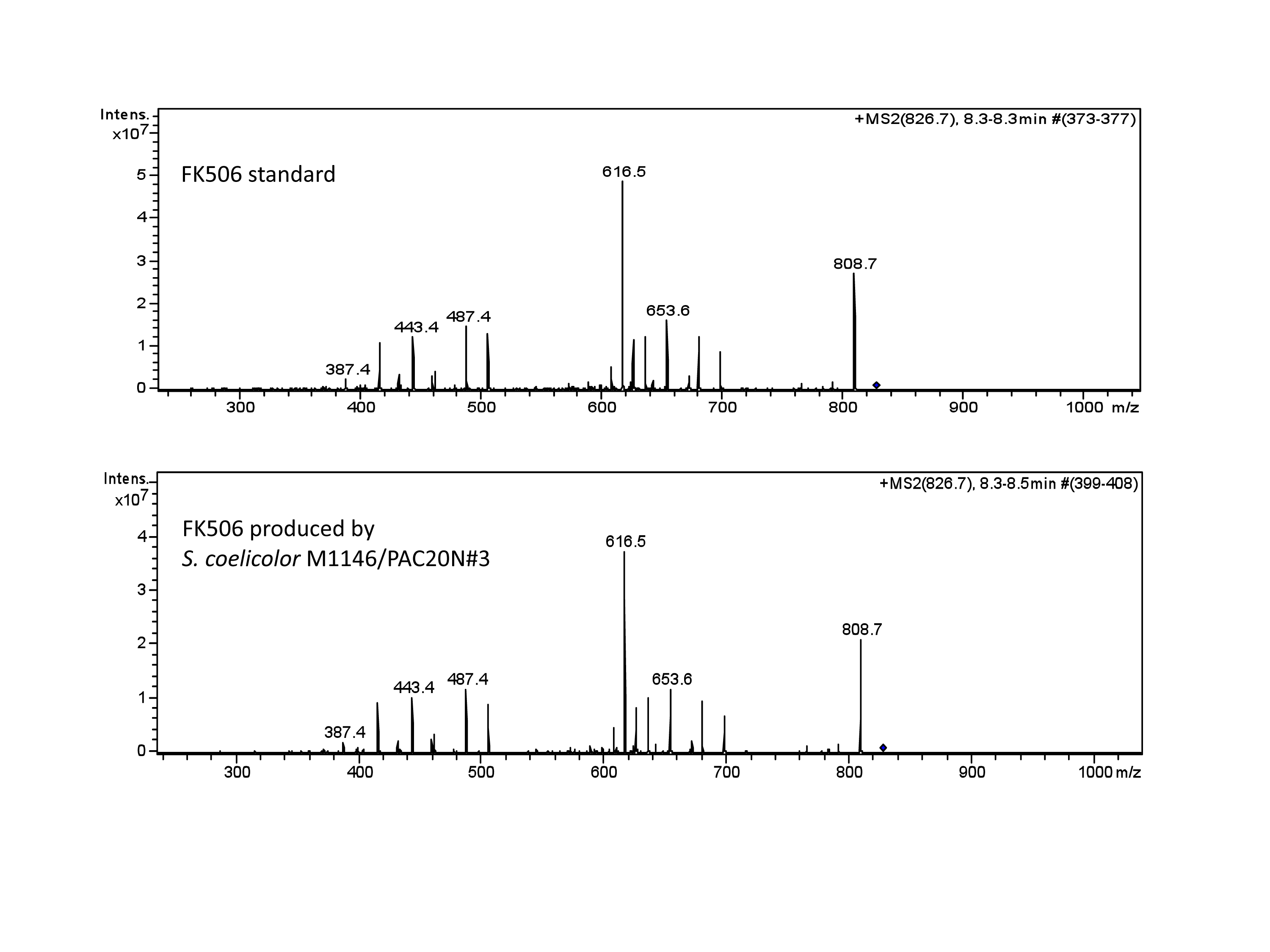

Supplement: Figure S1 — LCMS-MS analysis of FK506 standard (top) and FK506 produced by Streptomyces coelicolor M1146/PAC20N#3 (bottom). The parent ion is the sodium adduct of FK506 (826.7 m/z). (TIF) [file pone.0069319.s001.tif]
